# Negative legacy of obesity

**DOI:** 10.1371/journal.pone.0186303

**Published:** 2017-10-26

**Authors:** Kohsuke Shirakawa, Jin Endo, Yoshinori Katsumata, Tsunehisa Yamamoto, Masaharu Kataoka, Sarasa Isobe, Naohiro Yoshida, Keiichi Fukuda, Motoaki Sano

**Affiliations:** 1 Department of Cardiology, Keio University School of Medicine, Tokyo, Japan; 2 Department of Endocrinology and Hypertension, Tokyo Women’s Medical University, Tokyo, Japan; Niigata Daigaku, JAPAN

## Abstract

Obesity promotes excessive inflammation, which is associated with senescence-like changes in visceral adipose tissue (VAT) and the development of type 2 diabetes (T2DM) and cardiovascular diseases. We have reported that a unique population of CD44^hi^ CD62L^lo^ CD4^+^ T cells that constitutively express PD-1 and CD153 exhibit cellular senescence and cause VAT inflammation by producing large amounts of osteopontin. Weight loss improves glycemic control and reduces cardiovascular disease risk factors, but its long-term effects on cardiovascular events and longevity in obese individuals with T2DM are somewhat disappointing and not well understood. High-fat diet (HFD)-fed obese mice were subjected to weight reduction through a switch to a control diet. They lost body weight and visceral fat mass, reaching the same levels as lean mice fed a control diet. However, the VAT of weight reduction mice exhibited denser infiltration of macrophages, which formed more crown-like structures compared to the VAT of obese mice kept on the HFD. Mechanistically, CD153^+^ PD-1^+^ CD4^+^ T cells are long-lived and not easily eliminated, even after weight reduction. Their continued presence maintains a self-sustaining chronic inflammatory loop via production of large amounts of osteopontin. Thus, we concluded that T-cell senescence is essentially a negative legacy effect of obesity.

## Introduction

Weight loss is recommended for overweight and obese individuals with T2DM. Weight loss improves glycemic control and reduces cardiovascular disease risk factors, but epidemiological studies examining the long-term effects of weight reduction on cardiovascular events and the longevity of obese individuals with T2DM have yielded conflicting results. Meta-analysis showed that weight loss is associated with reduced mortality in unhealthy obese individuals [1]. Bariatric surgery reduces the incidence of myocardial infarction in obese individuals over a mean follow-up of 13.3 years in patients with T2DM [2]. However, a prospective randomized study (Look AHEAD trial) demonstrated that intensive lifestyle intervention focused on weight loss did not reduce cardiovascular morbidity and mortality in overweight or obese adults with T2DM at a median follow-up of almost 10 years [3].

Although chronic inflammation in visceral adipose tissue (VAT) is an important mediator in the development of T2DM and cardiovascular diseases in overweight and obese individuals, few studies have addressed the consequences of weight loss on VAT inflammation. Schmitz et al. demonstrated that VAT inflammation caused by obesity persisted after weight reduction [4], and this result may offer insight into a sustained increase in cardiovascular risk in obese individuals even after weight loss. However, why VAT inflammation persists despite a normalization of adipose tissue mass after weight loss remains unknown.

Functional changes in T cells associated with visceral obesity have been linked to the development of chronic inflammation in VAT. Work from our laboratory focuses on a unique population of CD44^high^ CD62L^low^ CD4^+^ T cells that constitutively express Programmed cell death 1 (PD-1) and CD153 and preferentially increase and accumulate in the VAT of HFD-fed obese mice. The CD153^+^ PD-1^+^ CD44^high^ CD62L^low^ subpopulation of CD4^+^ T cells showed markedly increased expression of senescence-associated β-galactosidase (SA-β-gal), γH2AX, and Cdkn1a/2b, suggestive of cellular senescence. These T cells also showed strong activation of *Spp1* encoding osteopontin (OPN) *in situ* in VAT, and they produced large amounts of OPN upon T-cell receptor stimulation *in vitro* in a PD-1–resistant manner. These features were highly reminiscent of senescence-associated CD4^+^ T cells that contribute to the changes in the immune system associated with age, a pattern known as immune-aging, which is characterized by impaired acquired immune capacity, increased pro-inflammatory traits, and high risk for autoimmunity [5] [6]. Remarkably, adoptive transfer of CD153^+^PD-1^+^CD44^hi^CD4^+^ T cells from HFD-fed WT, but not *Spp1*-deficient, mice into the VAT of lean mice fed a normal diet recapitulated the essential features of VAT inflammation and insulin resistance. Our results indicated that obesity accelerates T-cell senescence in VAT, and senescent CD153^+^PD-1^+^CD44^hi^CD4^+^ T cells play a key role in the development and maintenance of chronic inflammation of VAT by producing large amounts of OPN.

We hypothesized that CD153^+^PD-1^+^CD44^hi^CD4^+^ T cells in VAT, once present, remain for a long time, which offers a mechanistic basis of persistent VAT inflammation after weight reduction in obese subjects.

## Materials and methods

### Animal care

C57BL/6 (B6) mice were purchased from Clea Japan (Tokyo, Japan). All mice were housed under a 12-h light–dark cycle and allowed free access to food. Mice were fed with either a control diet (D12450J 10 kcal% fat) or a high-fat diet (HFD) (D12492, 60 kcal% fat, Research Diets). Mice were fed either a HFD starting at 4 weeks of age or kept on a control diet. At 30 weeks of age, half of the HFD-fed obese mice switched to the control diet. We analyzed mice at 38 weeks.

### Glucose and insulin tolerance tests

We performed glucose tolerance (oral administration of 1.5 g/kg of glucose, after 16 h of fasting) and insulin tolerance (administration of 0.75–2 U/kg of insulin intraperitoneally, after 4 h of fasting) tests to assess glucose intolerance and insulin resistance.

### Histologic analysis

To evaluate the size of adipocytes and the presence of lipid droplets, VAT and liver were obtained from mice fed control diet, HFD, and HFD switching to control diet. Serial sections were stained with hematoxylin-eosin and azan and were examined using software (BZ-H1C: Keyence, Osaka, Japan). Micrographs were taken from each section at, ×10 magnification with a digital camera (BIOREVO: Keyence).

### Immunohistochemical analysis

As previously reported [7], we stained and visualized whole-mount adipose tissue. Mice were sacrificed by cervical dislocation, after which the VAT was removed using sterile technique and minced into small pieces (~2–3 mm) using a scalpel. We washed the tissue pieces, which were then fixed in cellFIX (Cat. 340181, BD) for 60 min and permeabilized with 0.1% Triton X-100 for 10 min. The specimens were then blocked with 5% bovine serum albumin and incubated first with a primary antibody [F4/80 (BM-8, eBioscience)] overnight at 4°C and then with Alexa Fluor 488-conjugated secondary antibody (Molecular Probes) for 1 h. The tissues were counterstained for 1 h with BODIPY 558/568 (Molecular Probes) to visualize adipocytes and with DAPI (Molecular Probes) to visualize nuclei. We excited the tissue samples using four laser lines (405 nm, 488 nm, 568 nm, and 800 nm) and collected the emission through appropriate narrow band-pass filters in a confocal microscope (LSM 710, Carl Zeiss). The images were acquired and processed by LSM 710 software.

### Isolation of the stromal vascular fraction and flow cytometry

We isolated stromal vascular cells using previously described methods [7], with some modifications. Mice were sacrificed under general anesthesia after systemic heparinization. eVAT was removed and ground into small pieces. Samples were incubated for 40 min in collagenase II/DNase I solution (1 mg/ml collagenase II and 50 μg/ml in HBSS solution) with gentle stirring. Digested tissue was then centrifuged at 1000 *g* for 10 min. The resulting pellets were washed twice with cold PBS and filtered through a 70-mm mesh. Red blood cells were lysed with erythrocyte-lysing buffer (Biolegend) for 10 min and resuspended in RPMI-1640 supplemented with 10% FBS. Single-cell suspensions of adipose stromal vascular fraction (SVF) were blocked with CD16/32 monoclonal antibody (2.4G2; BD Biosciences) at 4°C for 5 min. Cells were stained with a mixture of antibodies at 4°C for 20 min. Flow cytometric analysis and sorting were performed on a FACSAriaIII instrument (BD Biosciences) and analyzed using the FlowJo software (Tree Star). Antibodies were specific to CD3 (14A-2; Biolegend), CD4 (GK1.5;Biolegend), CD8a (OKT8; Biolegend), CD44 (IM7; Biolegend), CD11b (M1/70; Biolegend), F4/80 (BM8; Biolegend), PD-1 (29F.1A12, Biolegend), CD19 (1D3, eBioscience), and CD153 (RM153, Biolegend)

### Real-time quantitative PCR

Total RNA samples from sorted cells, and adipose tissue were prepared using an RNeasy Mini Kit (Qiagen) or Trizol reagent (Invitrogen), according to the manufacturer’s instructions. A First-Strand cDNA Synthesis kit (Invitrogen) was used for cDNA synthesis. Quantitative real-time PCR was performed using ViiA™ 7 Real-Time PCR System. GAPDH gene was used as an endogenous control to normalize for differences in the amount of total RNA in each sample. All values were expressed as fold increase or decrease relative to the expression of GAPDH. Primer sequences for genes are as follows: Gapdh, 5′-AGGTCGGTGTGAACGGATTTG-3′ and 5′-TGTAGACCATGTAGTTGAGGTCA-3′; Spp1, 5’-CCCGGTGAAGTGCTGATT-3′ and 3′-TTCTTCAGAGGACACAGCATTC-3′; Ifng, 5′-ATCTGGAGGAACTGGCAAAA-3′ and 5′-TTCAAGACTTCAAAGAGTCTGAGG-3′; Tnf, 5′-CCCTCACACTCAGATCATCTTCT-3′ and 5′-GCTACGACGTGGGCTACAG-3′; Il1b, 5′-GCAACTGTTCCTGAACTCAACT-3′ and 5′- ATCTTTTGGGGTCCGTCAACT-3′; Il6, 5′-TAGTCCTTCCTACCCCAATTTCC-3′ and 5′-TTGGTCCTTAGCCACTCCTTC-3′; Col1a1, 5′-GCTCCTCTTAGGGGCCACT-3′ and 5′-CCACGTCTCACCATTGGGG-3′

### Cellular senescence assay

We isolated the SVF from the VAT and splenocytes of WT mice fed a HFD. SA-β-gal assays were performed using a cellular senescence live cell analysis kit as previously discribed [8].

### ELISA

Levels of OPN (R&D systems), total IgG (eBiosciences), and insulin (Morinaga) in supernatants or serum were determined by ELISA according to the manufacturers’ instructions.

### T-cell proliferation assay

3 × 10^5^ cells of PD-1^−^CD4^+^ CD44^+^ T cells, PD-1^+^CD153^-^CD4^+^CD44^+^ and PD-1^+^CD153^+^CD4^+^CD44^+^ T cells were sorted from spleen of 38-week-old HFD mice and cultured under IL-2 conditions for 15 days. Live or dead cells were analyzed by LIVE/DEAD Viability/Cytotoxicity Kit (Thermo Fisher) according to the manufacturers’ instructions.

### Statistical analysis

All values are presented as mean ± SEM. The statistical significance of differences between two groups was determined using a Student’s t-test. Differences among multiple groups were compared using ANOVA followed by Tukey’s tests.

### Study approval

This study conforms to the Guide for the Care and Use of Laboratory Animals published by the US National Institutes of Health (NIH publication no. 85–23, revised 1996), and it was approved by the Institutional Animal Care and Use Committee at the Keio University School of Medicine.

## Results and discussion

**Fig 1:**
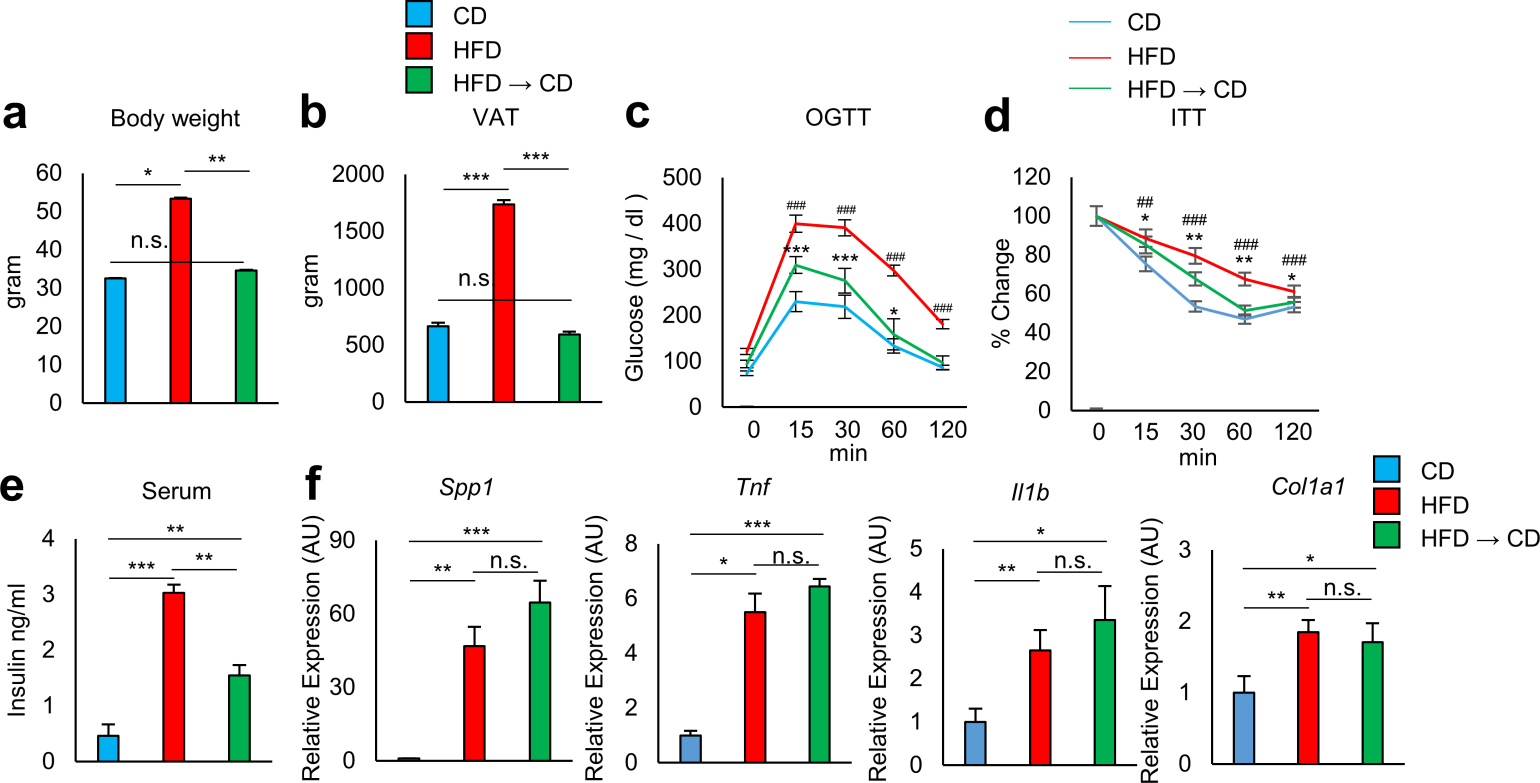
Effect of weight reduction on adipose tissue inflammation in obese mice. Mice were fed either a high-fat diet (HFD) or a control diet (CD). The HFD was introduced at 4 weeks of age. Half of the HFD mice were switched to control diet at 30 weeks of age (HFD to CD). The other half of the HFD mice were continuously kept on the HFD. These mice were evaluated at 38 weeks of age. **a, b** Body and eVAT weights were analyzed (n = 6 mice per group). **c, d** Effect of HFD or weight reduction on oral glucose tolerance test (OGTT) and insulin tolerance test (ITT) (n = 6 mice per group). *: CD vs HFD → CD, ^#^: CD vs HFD. **e** Serum insulin levels of mice fed CD, HFD, and HFD to CD (n = 3–9 mice per group). **f** Adipose tissue of indicated mice were analyzed for the expression of *Spp1*, *Tnf*, *Il1b*, and *Col1a1* by real-time PCR analysis (n = 6 mice per group). *P < 0.05, **P < 0.01, and ***P < 0.001, ^##^P < 0.01, and ^###^P < 0.001; n.s., not significant. Data are represented as mean ± SEM.

C57BL/6 (B6) mice were fed either a HFD (60 kcal% fat, D12492) starting at 4 weeks of age or kept on a control diet (10 kcal% fat, matching sucrose to D12492, D12450J). At 30 weeks of age, by which point the HFD-fed mice had developed severe obesity, half of the HFD-fed obese mice were switched to the control diet to induce weight reduction. The other half remained on the HFD. At 38 weeks of age, HFD-fed mice showed increased body weight and visceral fat mass, glucose intolerance, and insulin resistance compared to age-matched B6 mice fed a control diet (Fig 1A–1E). Weight reduction mice reached the same body weight as lean mice that had been maintained on the control diet (Fig 1A). VAT weight did not differ significantly between the weight reduction mice and the lean mice (Fig 1B); however, weight reduction did not fully improve glucose tolerance (Fig 1C) and insulin sensitivity (Fig 1D and 1E). Analysis of inflammation-related gene transcripts in VAT revealed that expression of *Spp1*, *Tnf*, *Il1b*, and *Col1a1* remained high in weight reduction mice to the same extent as in obese mice kept on the HFD (Fig 1F).

**Fig 2:**
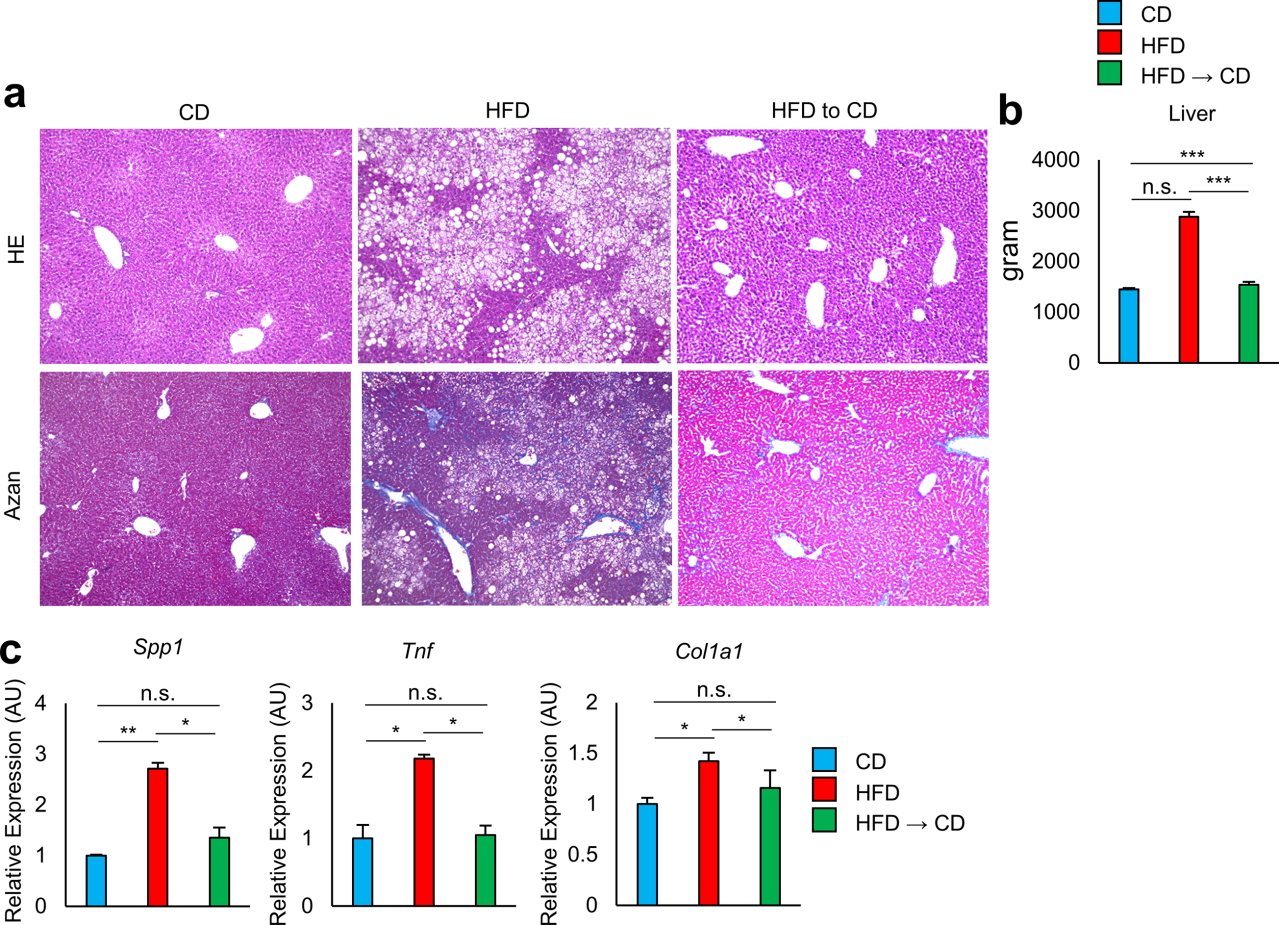
Effect of weight reduction on liver of obese mice. **a–c** Analysis of the livers of mice fed CD, HFD, and HFD to CD. **a** Livers were stained with hematoxylin-eosin (HE) or AZAN. Original magnification: ×10. Scale bar indicates 100μm. **b** Liver weights were analyzed (n = 6 mice per group). **c** Livers were analyzed for the expression of *Spp1*, *Col1a1*, and *Tnf* by real-time PCR analysis (n = 3–6 mice per group). *P < 0.05, **P < 0.01, and ***P < 0.001; n.s., not significant. Data are represented as mean ± SEM.

Obese mice kept on the HFD developed hepatic steatosis and fibrosis. Weight reduction caused disappearance of lipid droplets and improvement of fibrosis, and fully normalized the liver weight and the expression of *Spp1*, *Tnf*, and *Col1a1* to the same level as in the lean mice (Fig 2A–2C). These findings indicate that obesity-associated pro-inflammatory changes in the liver are quickly reversed in response to the decrease in fat deposits, while those in the VAT persist even after a decrease in excess fat.

It is widely accepted that, not only adipose tissue inflammation but also excessive inflammation of liver also has adverse effects on insulin resistance and glucose tolerance [9–11]. Weight reduction by shifting HFD to CD fully reversed hepatic steatosis. However, glucose metabolism was not fully resolved following weight normalization. We speculated that this is a result of residual adipose tissue inflammation.

**Fig 3:**
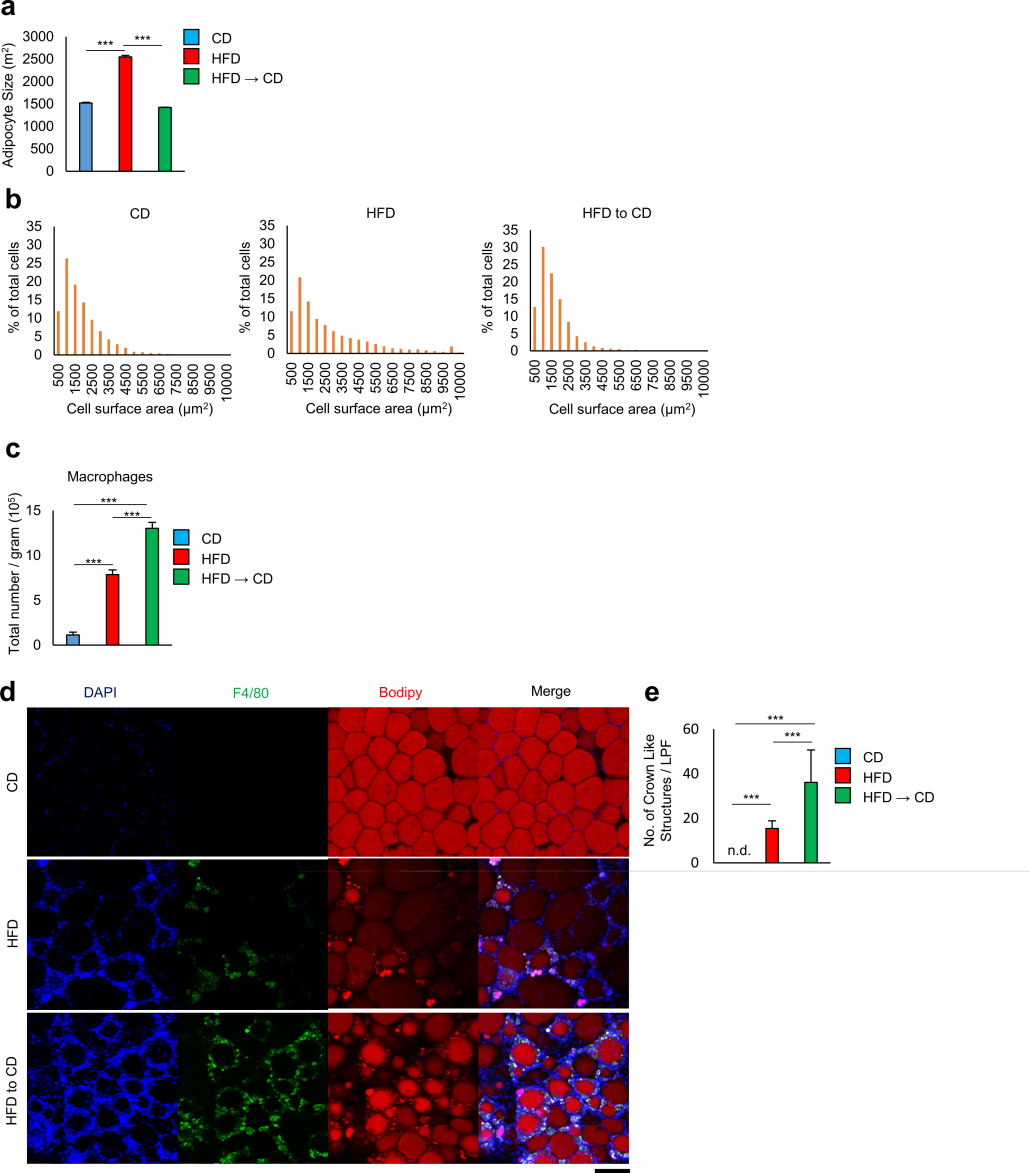
Effect of weight reduction on adipocyte size and crown-like strctures. **a** Quantitative measurements of adipocyte size (the average cross-sectional area of each adipocyte, μm^2^/cell) in VAT of mice fed CD, HFD, and HFD to CD (n = 3 mice per group). **b** Frequency distribution of adipocyte cell surface area from mice fed CD, HFD, and HFD to CD. **c** A flow cytometric analysis of cell number per gram of macrophages in visceral adipose tissue from mice fed CD, HFD, and HFD to CD (n = 3–5 mice per group). **d** Histochemical identification of adipocytes (BODIPY, red), F4/80 (yellow), and nuclei (DAPI, blue) in VAT. Scale bars, 100 μm. **e** Number of crown-like structures in adipose tissue of mice fed CD, HFD, and HFD to CD (n = 6 mice per group). ***P < 0.001; n.d., not detected. Data are represented as mean ± SEM.

Immunostaining analysis revealed that the VAT of obese mice kept on the HFD showed remarkably increased adipocyte size (Fig 3A and 3B) and increased numbers of F4/80^+^ macrophages that formed crown-like structures (CLSs), representing typical obesity-associated chronic inflammation of VAT (Fig 3C–3E). Notably, despite a normalization of fat cell size, the VAT of weight reduction mice exhibited denser infiltration of macrophages forming more CLSs compared to the VAT of obese mice kept on HFD.

In the early stage of weight loss, adipose tissue lipolysis drives adipose tissue macrophage recruitment without being accompanied by increases in adipose tissue inflammation and insulin resistance. However, lipolysis-promoted macrophage accumulation decreased after prolonged periods of weight reduction[12]. As we confirmed in the latter part of the two months, accumulation of macrophages at this time seems not to just reflect extensive remodeling of adipose tissue.

**Fig 4:**
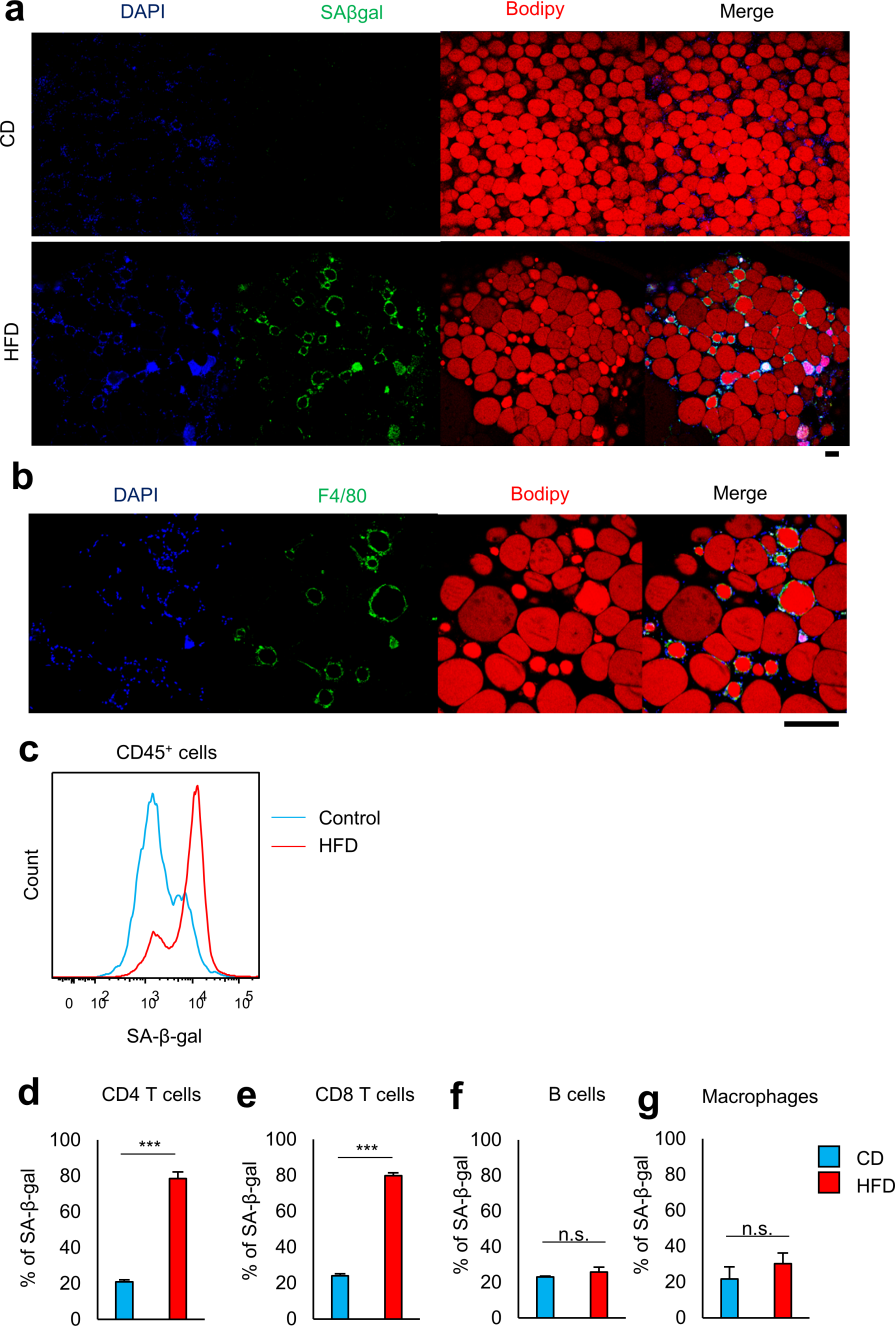
**Visceral fat accumulation promotes senescence-like changes in VAT, a, b** Histochemical identification of adipocytes (BODIPY, red), SA-β-gal (yellow), and nuclei (DAPI, blue) in VAT. Scale bars, 100 μm. Original magnification: ×10 (a), ×40 (b). **c** A representative flow cytometric analysis demonstrating SA-β-gal activity of CD45^+^ T cells in the VAT of mice fed CD or HFD (n = 3 mice per group). **d–g** Flow cytometric analysis of SA-β-gal activity on CD4^+^ T cells, CD8^+^ T cells, B cells, and macrophages from VAT (n = 3–5 mice per group). ***P < 0.001; n.s., not significant. Data are represented as mean ± SEM.

Visceral fat accumulation promotes senescence-like changes in VAT, such as increased activity of SA-β-gal [13] [14]. No apparent SA-β-gal activity was detected in adipocytes in obese mice kept on the HFD, while cells surrounding the adipocytes were positive for SA-β-gal (Fig 4A and 4B). Flow cytometric analysis confirmed that SA-β-gal^+^ nonadipocytes were CD45^+^ immune cells (Fig 4C). Around 80% of VAT CD4^+^ and CD8^+^ T cells in obese mice were SA-β-gal positive, whereas those in lean control mice were minimally positive (Fig 4D and 4E and S1A and S1B Fig). The proportion of cells positive for SA-β-gal activity in other infiltrated cell populations such as macrophages and B cells was not altered in obese adipose tissue (Fig 4F and 4G and S1C and S1D Fig). These findings suggest that VAT T cells are prone to senescence in response to excess energy intake.

**Fig 5:**
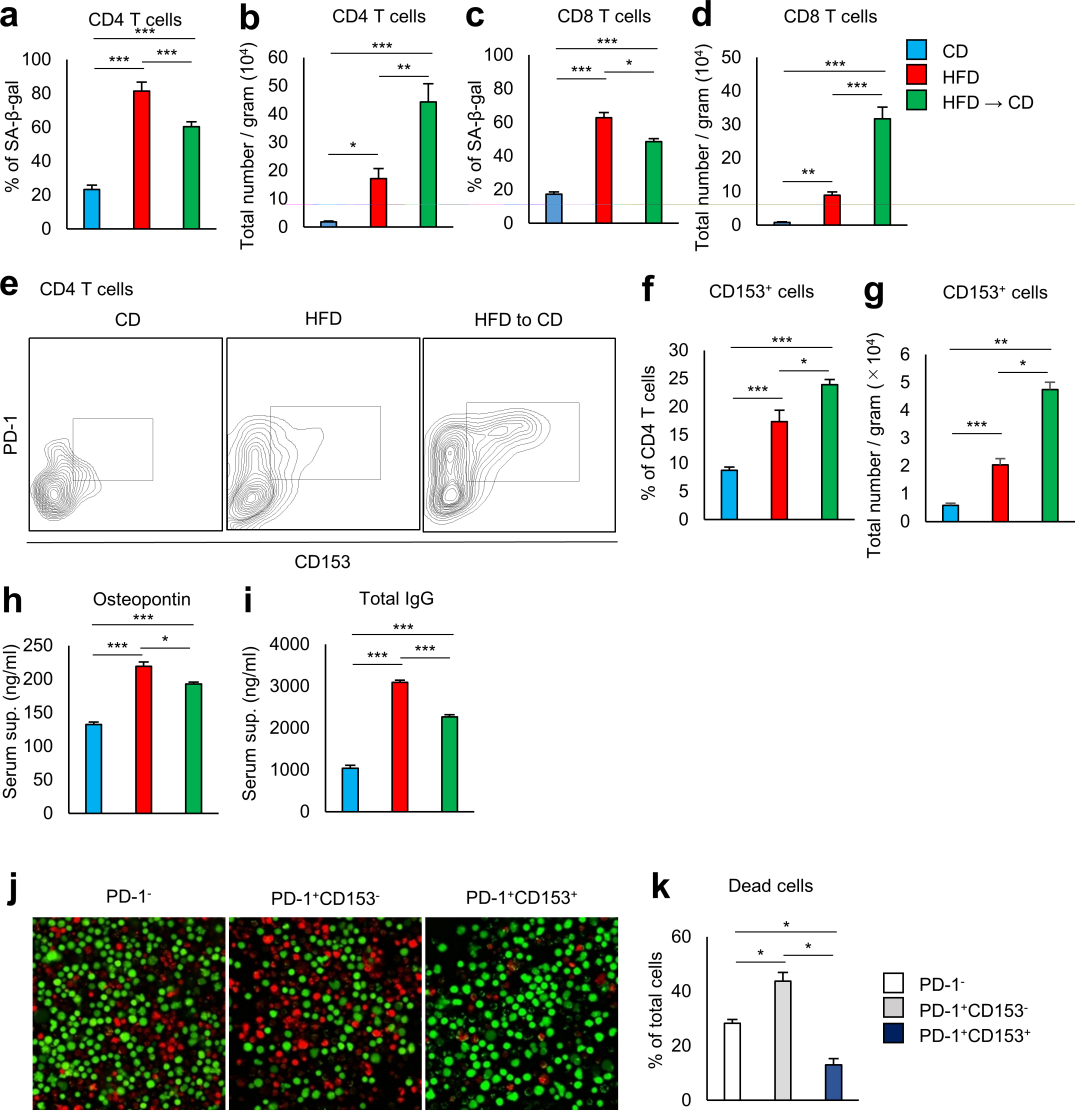
Effect of weight reduction on T lymphocytes in VAT of obese mice. **a–d** Flow cytometric analysis of SA-β-gal activity on CD4^+^ T cells and CD8^+^ T cells from VAT. (n = 5 mice per group). **a, c** Expression of SA-β-gal on CD4^+^ T cells and CD8^+^ T cells in the VAT of mice fed CD, HFD, and HFD to CD. **b, d** Results are expressed as cell number per gram of SA-β-gal^+^ cells. **e–g** Flow cytometric analysis of VAT CD153^+^ PD-1^+^ CD4^+^ T cells obtained from mice fed CD, HFD, and HFD to CD. **e** A representative flow cytometric analysis of VAT CD153^+^ PD-1^+^ CD4^+^ T cells. **f** Expression of CD153^+^ on CD4^+^ T cells. **g** Results are expressed as cell number per gram of CD153^+^ PD-1^+^ CD4^+^ T cells (n = 5–10 mice per group). **h, i** Serum osteopontin (**h**) and total IgG (**i**) from mice fed CD, HFD, and HFD to CD were assessed by ELISA (n = 5–8 mice per group). **j, k** PD-1^−^, CD153^-^ PD-1^+^, CD153^+^ PD-1^+^ CD4^+^ T cells were separately isolated from the spleens of obese mice. Indicated cells were cultured with IL-2 for 15 days and analyzed for live and dead cells (n = 3–4 mice per group). *P < 0.05, **P < 0.01, and ***P < 0.001. Data are represented as mean ± SEM.

We then examined the effects of weight loss on VAT T-cell senescence. The proportion of VAT CD4^+^ T cells positive for SA-β-gal activity remained high in weight reduction mice (Fig 5A and S2 Fig), and the numbers of SA-β-gal ^+^ CD4^+^ T cells per gram of VAT was even higher than in obese mice kept on HFD (Fig 5B). A similar change was observed in VAT CD8^+^ T cells (Fig 5C and 5D).

Recently, we found that CD153 expression defines a unique CD4^+^ T-cell population with features of cell senescence that increases almost exclusively in the VAT of HFD-fed mice [8]. Senescent T cells were barely seen in the VAT of lean mice kept on the control diet. These cells play a crucial role in VAT inflammation by producing large amounts of OPN. The proportion of VAT CD4^+^ T cells positive for CD153 in weight reduction mice was somewhat more than in obese mice kept on the HFD (Fig 5E and 5F). The numbers of CD153^+^ CD4^+^ T cells per gram of VAT in weight reduction mice were more than twice those seen in obese mice kept on HFD (Fig 5G).

Using EGFP-*Spp1* KI reporter mice, we demonstrated that a significant proportion of CD153^+^ PD-1^+^ CD44^high^ CD4 T cells *in situ* in the VAT of HFD-fed mice showed a remarkable activation of *Spp1 in situ* that was far greater than that found in macrophages [8]. Adoptive transfer of CD153^+^PD-1^+^CD44^hi^CD4^+^ T cells from HFD-fed WT, but not *Spp1*-deficient, mice into the VAT of lean mice fed a normal diet recapitulated the essential features of VAT inflammation and insulin resistance and exhibited a significant increase in serum OPN and IgG levels to the same extent as observed in HFD-fed obese mice. Consistent with this finding, weight reduction mice continued to have high serum OPN and IgG levels despite a reduction in total VAT size (Fig 5H and 5I). It is a problem that the blood concentration of OPN is maintained high even after weight reduction. OPN acts as a bad substance to accelerate aging via stimulating inflammatory response and promoting organ remodeling [15–18].

To understand the mechanism behind survival and accumulation of CD153^+^ CD4^+^ T cells in VAT after reduction of visceral fat, we compared the resistance to cell death, using three different subsets of CD4^+^ T cells. T cells cause cell death upon prolonged culture with T-cell receptor stimulation and IL-2. CD153^+^ CD4^+^ T cells were more resistant to cell death than CD153^−^ subsets (Fig 5J and 5K).

We assumed that disease-specific cell populations that do not exist in normal circumstances are involved in the development of chronic inflammation. We found that senescent T cells prematurely emerged and increased in proportion to the amount of visceral adiposity. This discovery may provide the insight needed to develop effective treatments to stem the progression of cardiovascular and metabolic complications associated with obesity. Eliminating the senescent T cells from the body is a key point in the success of such treatments. However, we emphasize that preventing excessive weight gain is important. Senescent T cells, once they are present cannot be easily eliminated. As long as senescent T cells remain, VAT inflammation persists. We conclude that senescent T cells are essentially a negative legacy effect of obesity.

## Supporting information

S1 Fig Effect of HFD on adipose senescence. **a-d** A representative flow cytometric analysis demonstrating SA-β-gal activity of CD4^+^ T cells, CD8^+^ T cells, B cells, and macrophages in the VAT of mice fed CD or HFD (n = 3–5 mice per group).
(TIF)

S2 Fig Effect of weight reduction on adipose senescence of obese mice. A representative flow cytometric analysis demonstrating SA-β-gal activity of CD4^+^ T cells, CD8^+^ T cells in VAT of mice fed CD, HFD, and HFD to CD (n = 3–5 mice per group).
(TIF)
