# Supplementary figures and images for: Negative legacy of obesity
Source: PLoS One. 2017 Oct 26;12(10):e0186303. doi: 10.1371/journal.pone.0186303 (PMC5657997; doi:10.1371/journal.pone.0186303)

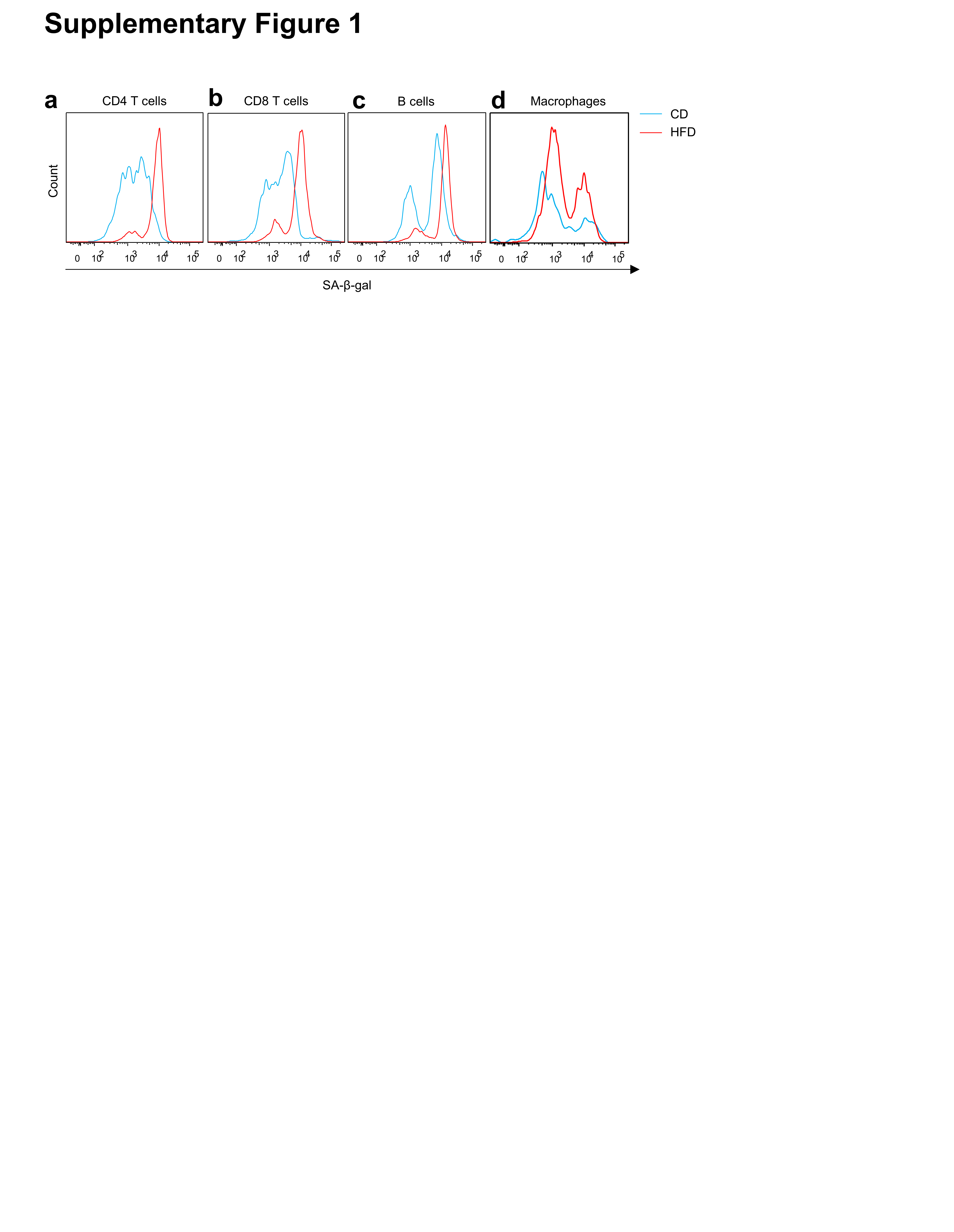

Supplement: S1 Fig — a-d A representative flow cytometric analysis demonstrating SA-β-gal activity of CD4+ T cells, CD8+ T cells, B cells, and macrophages in the VAT of mice fed CD or HFD (n = 3–5 mice per group). (TIF) [file pone.0186303.s001.TIF]

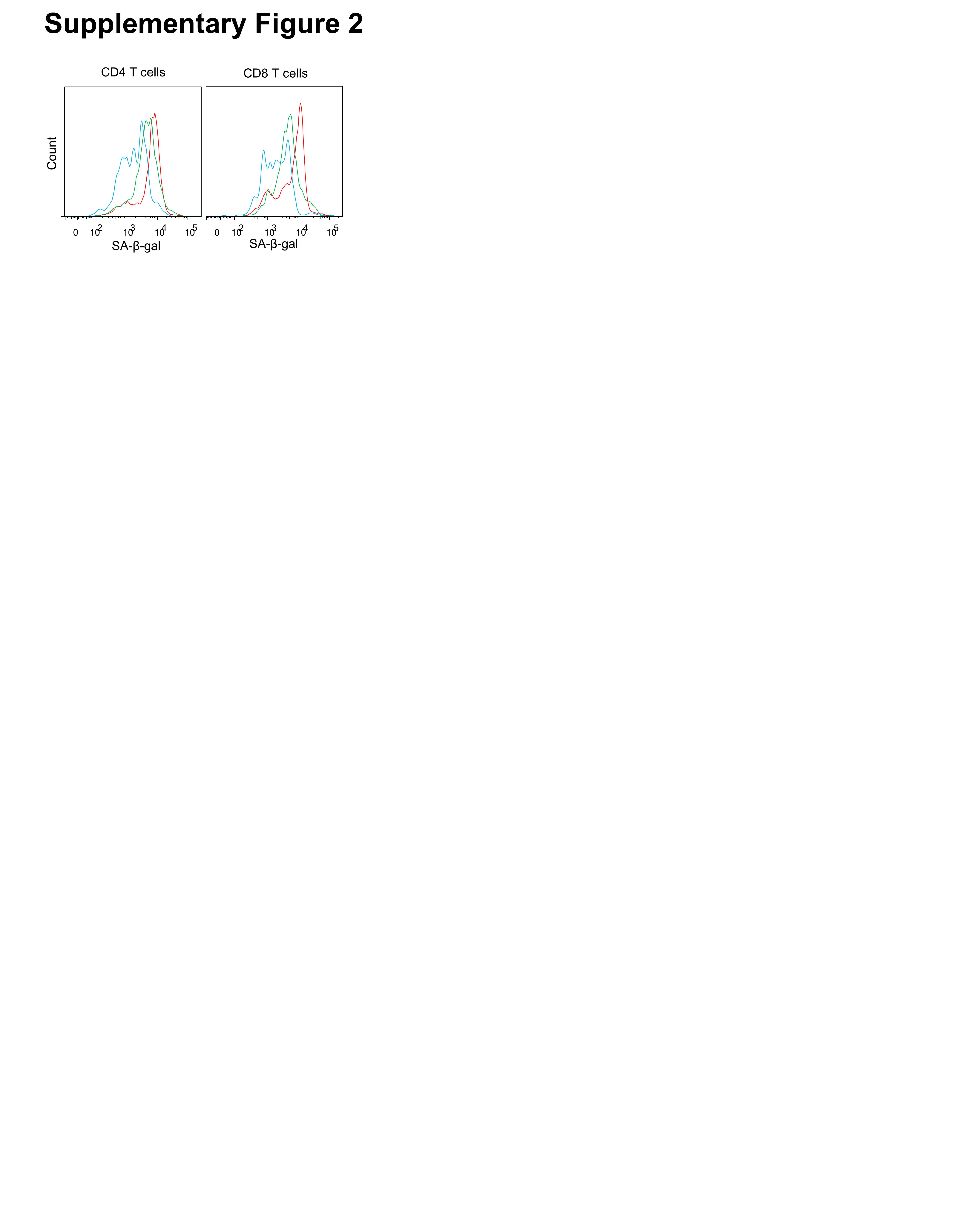

Supplement: S2 Fig — A representative flow cytometric analysis demonstrating SA-β-gal activity of CD4+ T cells, CD8+ T cells in VAT of mice fed CD, HFD, and HFD to CD (n = 3–5 mice per group). (TIF) [file pone.0186303.s002.TIF]
